# Exploring mechanisms behind the increasing gender gap in adolescent psychological symptoms, 2002–2022: the role of national‐level gender equality

**DOI:** 10.1111/jcpp.14081

**Published:** 2024-12-07

**Authors:** Margreet E. de Looze, Alina Cosma, Frank J. Elgar, Karen Schrijvers, Jo Inchley, Sophie D. Walsh, Gonneke W. J. M. Stevens

**Affiliations:** ^1^ Department of Interdisciplinary Social Science, Faculty of Social and Behavioural Sciences Utrecht University Utrecht The Netherlands; ^2^ Department of Sociology Trinity College Dublin Dublin Ireland; ^3^ School of Psychology Trinity College Dublin Dublin Ireland; ^4^ Department of Equity, Ethics and Policy, School of Population and Global Health McGill University Montreal QC Canada; ^5^ Department of Public Health and Primary Care, Faculty of Medicine and Health Sciences Ghent University Ghent Belgium; ^6^ MRC/CSO Social and Public Health Sciences Unit University of Glasgow Glasgow UK; ^7^ Department of Criminology Bar Ilan University Ramat Gan Israel

**Keywords:** Adolescence, gender differences, mental health, cross‐cultural, public health

## Abstract

**Background:**

Internalising problems have increased considerably among adolescents in the last decades, particularly among girls, resulting in widening gender gaps. This study examined whether the gender gap in psychological symptoms increased more in more gender‐equal countries in the period 2002–2022, and if so, to what extent this could be explained by changes over time in the experience of stressors (i.e. schoolwork pressure, body dissatisfaction, low classmate support) among boys and girls in these countries.

**Methods:**

National data on gender inequality (UNDP Gender Inequality Index) were combined with aggregated individual‐level data from the Health Behaviour in School‐aged Children (HBSC) study (2002–2022) across 43 countries (*N* = 1,268,220). Absolute and relative gender gaps in psychological symptoms were regressed on survey cycle, GII and their interaction. Next, interactions of survey cycle and either schoolwork pressure, body dissatisfaction or classmate support were added to the model.

**Results:**

Increases in the absolute and relative gender gap in psychological symptoms between 2002 and 2022 were stronger in more gender‐equal countries, mainly due to larger increases in psychological symptoms among girls in these countries. Also, less favourable time trends for schoolwork pressure and classmate support were found in more gender‐equal countries for boys and especially girls. The larger increase in schoolwork pressure among girls in more gender‐equal countries partly explained the increased absolute gender gap in psychological symptoms in these countries.

**Conclusions:**

While national‐level gender equality was positively associated with boys' and girls' mental health in the early 2000s, this association has become negative for girls in more recent years. The benefits of gender equality for girls' mental health may have become overshadowed by the increased experience of stressors, especially schoolwork pressure. Far from advocating that gender equality is a negative situation, these findings suggest that much work remains to achieve full gender equality, where men and women really share the burdens and stressors in everyday life.

One of the most consistent findings in the field of adolescent mental health is the gender gap, with girls reporting higher levels of internalising problems than boys (e.g. Chen, Cai, He, & Fan, 2020; Cosma et al., 2023; Salk, Hyde, & Abramson, 2017). As the adolescent years are a critical stage for lifelong mental health (Kessler et al., 2007), the gender gap in internalising problems among adolescents sets the stage for the higher prevalence of mental health disorders in adult women compared to men worldwide (Albert, 2015). Recent evidence suggests that internalising problems in adolescents have increased considerably over time across many countries (Boer et al., 2023; Cosma et al., 2020), and more so among girls than boys (Cosma et al., 2023), resulting in an even larger gender gap. Cross‐country differences are however considerable, making it imperative to understand not only which factors have negatively impacted the mental health of girls in particular, but also in which national contexts these trends were most likely to take place.

National‐level gender equality, defined as equal rights, responsibilities and opportunities in society for women and men, girls and boys (UNICEF, 2011), may be a societal factor that (partly) explains the cross‐national variety in the trends in the gender gap in adolescent internalising problems (Guo et al., 2022). Theoretically, two contrasting hypotheses can be formulated on the association between national‐level gender (in)equality and trends over time in these gender gaps. On the one hand, in more gender‐equal countries, girls in particular may have more development opportunities and may be less restricted by gender norms, as compared to girls in gender‐unequal countries (Greene & Patton, 2020). Throughout history, girls and women have faced systematic discrimination and unequal opportunities in society (Chant, 2016); gender equality seeks to address and rectify these disadvantages, ensuring that girls and women have equal access to education, healthcare, employment and other opportunities (Hearn & Husu, 2016). Moreover, gender equality can empower girls by challenging traditional gender norms and by providing them with the agency to make informed decisions about the future, contributing to their personal development and well‐being (Heise et al., 2019). While national‐level gender equality also challenges traditional gender norms and provides more opportunities for boys (Holter, 2014), which may improve their well‐being as well (de Looze, Huijts, Stevens, Torsheim, & Vollebergh, 2018), girls can be theorised to benefit more from it due to their historically disadvantaged position. Consequently, we may expect that over‐time increases in the gender gap in adolescent internalising problems may be *smaller* in more gender‐equal countries.

On the other hand, living in a more gender‐equal society may generate a double burden on girls (Zuckerman, Li, & Diener, 2017): girls may perceive higher expectations regarding their educational and professional development (Curran & Hill, 2022; Högberg, Strandh, & Hagquist, 2020), while at the same time, they may still feel pressure to adhere to roles that are traditionally seen as ‘female’, such as providing emotional support to their peers and having high beauty standards (Zuckerman et al., 2017). Together, these burdens may lead adolescent girls in more gender‐equal countries to perceive relatively high levels of stressors, in particular, schoolwork pressure and to feel less satisfied with their bodies and their relationships with peers (Högberg et al., 2020; Whitehead et al., 2017). Among boys, in contrast, more gender‐equal environments may *not* increase the pressure to succeed academically and professionally and to adhere to masculine beauty standards (i.e. be strong and muscular) to prove their masculinity, as they may feel they can ‘share the burden’ with girls (Oliffe et al., 2023). As schoolwork pressure, body dissatisfaction and negative social relationships with peers are all associated with increased internalising problems (Cosma et al., 2020; Viner et al., 2012), it can be expected that in more gender‐equal countries, over‐time increases in internalising problems may be more substantive for girls, but less so for boys. As a result of this, the gender gap in adolescent internalising problems can be expected to be *larger* in more gender‐equal countries.

Empirical studies on the association between national‐level gender inequality and the gender gap in adolescent mental health show contrasting findings. Studies using data from the late 1990s and early 2000s showed that both genders reported lower psychological symptoms (Torsheim et al., 2006), higher life satisfaction (de Looze et al., 2018) and better health outcomes overall (Viner et al., 2012) if they lived in more gender‐equal countries. Thus, according to these studies, both genders seemed to benefit from national‐level gender equality to an similar extent, suggesting no direct link between national‐level gender equality and the gender gap in adolescent mental health. However, a more recent study (Campbell, Bann, & Patalay, 2021), based on 2018 data across more than 70 countries, reported *lower* levels of mental health among boys and girls in more gender‐equal countries and *larger* gender gaps across a wide range of mental health outcomes, as compared to adolescents in gender‐unequal countries. Also, based on 2015 and 2018 data, Guo et al. (2022) showed that national‐level gender equality was associated with a *larger* gender gap in adolescent subjective well‐being, resulting from higher subjective well‐being among boys, but not girls, in more gender‐equal countries. Finally, a study based on 2018 data found that gender differences in life satisfaction, school pressure, multiple health complaints, and feeling fat were larger in more gender‐equal countries (Heinz, Catunda, Duin, Torsheim, & Willems, 2020).

Combining the empirical evidence from recent years, the literature suggests that the impact of national‐level gender equality on boys' and girls' mental health might have changed over time, leading to larger over‐time increases in the gender gap in adolescent mental health in more gender‐equal countries.

## Current study

**Figure 1:**
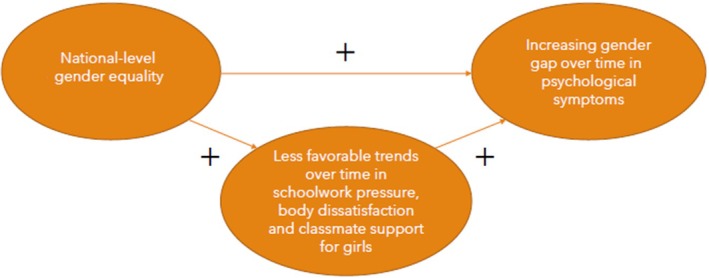
Conceptual model used in this paper

Based on the combination of the theoretical reasoning and available empirical research outlined above, we hypothesised that the benefits of national‐level gender equality for adolescent – and in particular girls' – mental health (as observed in the early 2000s) in recent years may have become overshadowed by increased stressors placed on girls (the ‘double burden’) (also see Figure 1). To test this hypothesis, we formulated the following research questions:

1. Is national‐level gender equality associated with increases in the gender gap in psychological symptoms between 2002 and 2022? If so, to what extent is this due to time trends in psychological symptoms among girls and/or boys in these countries?
2. To what extent is national‐level gender equality associated with larger increases in schoolwork pressure and body dissatisfaction and larger declines in classmate support between 2002 and 2022 among girls and/or boys?
3. If the gender gap in psychological symptoms has increased more in more gender‐equal countries, then to what extent can this be explained by changes over time in the experience of social stressors (i.e. more schoolwork pressure and body dissatisfaction and lower classmate support) among boys and girls in these countries?

## Methods

### Sample

In this study, UNDP national‐level data on gender inequality and World Bank data on country wealth were combined with aggregated individual‐level data from the internationally comparative Health Behaviour in School‐aged Children (HBSC) study (survey cycles 2002, 2006, 2010, 2014, 2018 and 2022). HBSC is an international survey on adolescent health, well‐being and the social context of health that is carried out in the classroom setting. The survey has taken place every 4 years since 1983 in a growing number of largely European and North American countries (Inchley, Stevens, Samdal, & Currie, 2020). The inclusion of HBSC data from 2002 onwards was informed by the need to have a large enough sample size at country level (more than 30 units) in order to run the multilevel modelling which was only possible after this time point. Additionally, some of the mediators tested in RQ3 (i.e. body dissatisfaction, classmate support) have been amended between 1998 and 2022 survey. In each survey cycle, stratified samples of schools, representing the regional, economic, and public–private distribution of schools in each country, were recruited according to a common protocol (Inchley et al., 2023). Each participating country obtained ethical approval to conduct the survey from their ethics review board or equivalent regulatory institution. Participation was voluntary and anonymous, and consent was obtained from school administrators, parents, and children, as per national human participant requirements. In total, 43 countries were included in the analysis for the current study (for an overview of the countries, see Table 1). Separate HBSC studies were carried out in Belgium (Flanders and Wallonia) and the UK (England, Scotland and Wales). Countries were included if they were involved in at least three survey cycles. The total sample included 1,268,220 (51% girls) adolescents aged 11–15 years old, in 234 country/year samples.

**Table 1:** Characteristics of the sample across survey years and countries (2002–2022) (*N* = 1,268,220)

Country	*n*	Cycle years	3–4 psychological symptoms per week	Some or a lot of schoolwork pressure	Body dissatisfaction	High classmate support	Gender Inequality Index, mean (*SD*)	GDP ($000 s), mean (*SD*)
Albania	12,243	14/18/22	2,763 (23.7%)	4,417 (36.4%)	529 (4.4%)	9,391 (77.1%)	0.172 (0.040)	12.8 (1.9)
Austria	27,128	02/06/10/14/18/22	4,798 (18.0%)	5,906 (22.3%)	2,128 (7.9%)	17,484 (65.4%)	0.096 (0.033)	45.3 (9.7)
Armenia	15,575	10/14/18/22	3,259 (24.8%)	4,182 (28.4%)	924 (6.0%)	11,130 (76.7%)	0.269 (0.056)	11.7 (2.9)
Belgium (Fl)	33,073	02/06/10/14/18/22	6,901 (21.3%)	9,942 (31.1%)	2,435 (7.5%)	20,320 (63.4%)	0.080 (0.031)	44.3 (10.2)
Belgium (Fr)	30,227	02/06/10/14/18/22	8,611 (29.6%)	9,174 (31.7%)	2,224 (7.6%)	17,502 (60.3%)	0.079 (0.029)	43.9 (9.3)
Bulgaria	17,204	06/10/14/18/22	4,783 (28.8%)	5,481 (33.5%)	1,277 (7.6%)	6,539 (40.2%)	0.227 (0.014)	18.7 (5.9)
Canada	64,677	02/06/10/14/18/22	16,395 (26.3%)	28,081 (44.2%)	3,470 (5.6%)	29,067 (45.6%)	0.104 (0.029)	42.6 (4.9)
Croatia	31,875	02/06/10/14/18/22	7,587 (24.7%)	9,537 (30.4%)	1,548 (4.9%)	19,235 (61.3%)	0.133 (0.028)	22.1 (5.9)
Czechia	43,771	02/06/10/14/18/22	13,703 (32.5%)	15,714 (36.2%)	3,459 (8.0%)	17,029 (39.4%)	0.134 (0.016)	34.9 (9.5)
Denmark	27,070	02/06/10/14/18/22	5,586 (21.2%)	7,560 (28.3%)	1,683 (6.3%)	17,796 (66.5%)	0.041 (0.018)	45.7 (11.7)
Estonia	26,343	02/06/10/14/18/22	7,633 (29.3%)	11,121 (42.5%)	2,252 (8.6%)	13,773 (52.6%)	0.166 (0.057)	26.9 (10.5)
Finland	29,953	02/06/10/14/18/22	6,877 (29.0%)	12,722 (43.4%)	2,069 (7.0%)	16,427 (55.6%)	0.066 (0.019)	39.7 (8.1)
France	41,641	02/06/10/14/18/22	11,621 (28.9%)	10,477 (25.4%)	2,273 (5.5%)	21,556 (52.3%)	0.122 (0.038)	38.2 (7.6)
Germany	34,712	02/06/10/14/18/22	6,246 (18.3%)	9,009 (26.7%)	3,374 (9.9%)	24,376 (71.7%)	0.092 (0.018)	42.7 (10.4)
Greece	26,720	02/06/10/14/18/22	9,304 (35.6%)	10,457 (39.5%)	2,676 (10.1%)	11,909 (44.7%)	0.148 (0.034)	26.9 (2.2)
Hungary	24,352	02/06/10/14/18/22	7,741 (32.6%)	5,599 (23.3%)	1,910 (8.1%)	12,897 (53.7%)	0.242 (0.020)	24.3 (7.5)
Iceland	48,155	02/06/10/14/18/22	13,801 (29.4%)	22,648 (48.2%)	2,243 (5.5%)	31,128 (65.6%)	0.073 (0.026)	45.2 (6.6)
Ireland	24,289	06/10/14/18/22	5,483 (23.6%)	9,501 (39.8%)	1,130 (4.8%)	14,953 (62.7%)	0.137 (0.045)	58.5 (24.2)
Israel	29,387	02/06/10/14/18	11,655 (42.7%)	9,975 (39.0%)	2,625 (9.5%)	18,768 (64.9%)	0.135 (0.035)	31.6 (5.6)
Italy	25,962	02/06/10/14/18/22	11,184 (43.6%)	12,684 (49.3%)	1,283 (5.0%)	15,797 (61.2%)	0.108 (0.048)	36.6 (5.3)
Latvia	27,899	02/06/10/14/18/22	8,166 (30.1%)	8,420 (30.5%)	1,932 (7.0%)	10,500 (45.7%)	0.213 (0.047)	23.0 (8.6)
Lithuania	31,091	02/06/10/14/18/22	9,040 (29.7%)	15,968 (52.3%)	2,090 (6.8%)	13,822 (45.1%)	0.159 (0.045)	24.3 (11.3)
Luxembourg	20,235	06/10/14/18/22	6,536 (33.0%)	6,482 (32.7%)	1,899 (9.6%)	13,065 (65.3%)	0.084 (0.032)	101.1 (17.0)
Malta	11,632	02/06/14/18/22	3,931 (35.6%)	6,708 (59.3%)	699 (6.1%)	7,377 (64.9%)	0.205 (0.044)	35.3 (10.3)
Moldova	14,825	14/18/22	3,654 (25.0%)	4,027 (27.2%)	715 (4.8%)	8,874 (59.9%)	0.230 (0.040)	10.9 (2.4)
Netherlands	26,452	02/06/10/14/18/22	4,724 (18.2%)	5,802 (22.5%)	1,240 (4.7%)	17,824 (69.3%)	0.050 (0.026)	48.1 (9.4)
North Macedonia	26,323	02/06/10/14/18/22	4,651 (22.5%)	11,079 (42.4%)	968 (4.5%)	19,797 (75.6%)	0.172 (0.037)	12.1 (4.1)
Norway	23,885	02/06/10/14/18/22	5,081 (22.0%)	7,467 (32.5%)	1,077 (6.4%)	15,744 (69.4%)	0.057 (0.027)	56.1 (10.6)
Poland	31,298	02/06/10/14/18/22	9,897 (32.2%)	13,100 (42.6%)	3,753 (12.1%)	12,634 (49.4%)	0.143 (0.022)	23.3 (9.6)
Portugal	27,192	02/06/10/14/18/22	5,655 (21.2%)	12,703 (47.8%)	1,385 (5.1%)	18,193 (68.2%)	0.107 (0.042)	29.2 (5.2)
Romania	27,017	06/10/14/18/22	8,748 (33.8%)	9,913 (38.0%)	1,440 (5.4%)	15,288 (58.3%)	0.317 (0.033)	24.9 (9.8)
Russia	30,439	02/06/10/14/18	6,362 (21.6%)	8,738 (29.4%)	1,185 (3.9%)	12,667 (42.7%)	0.324 (0.060)	17.5 (7.2)
Slovakia	25,694	06/10/14/18/22	7,629 (30.9%)	6,218 (25.2%)	1,628 (6.4%)	11,658 (47.4%)	0.189 (0.010)	27.9 (5.0)
Slovenia	31,513	02/06/10/14/18/22	6,935 (22.3%)	16,023 (51.2%)	1,818 (5.8%)	21,048 (67.1%)	0.099 (0.029)	31.7 (7.8)
Spain	39,318	02/06/10/14/18/22	7,104 (19.1%)	18,840 (50.4%)	2,363 (6.3%)	24,500 (65.0%)	0.096 (0.027)	31.8 (4.3)
Sweden	31,323	02/06/10/14/18/22	10,477 (34.5%)	9,785 (31.8%)	1,800 (5.9%)	20,979 (68.1%)	0.043 (0.011)	44.8 (8.4)
Switzerland	37,263	02/06/10/14/18/22	10,727 (29.4%)	9,613 (26.2%)	2,036 (5.6%)	25,862 (70.7%)	0.048 (0.024)	58.8 (11.4)
Turkey	17,151	06/10/18	8,239 (50.1%)	8,954 (52.6%)	1,037 (6.1%)	8,751 (51.6%)	0.408 (0.096)	19.7 (6.0)
Ukraine	26,261	02/06/10/14/18	7,913 (31.5%)	7,160 (28.2%)	1,035 (4.0%)	12,151 (47.7%)	0.305 (0.036)	9.2 (2.4)
UK: England	27,361	02/06/10/14/18/22	7,657 (29.1%)	12,646 (48.0%)	1,649 (6.3%)	13,361 (50.4%)	0.161 (0.043)	37.5 (6.1)
UK: Scotland	32,672	02/06/10/14/18/22	7,875 (24.6%)	11,902 (37.6%)	1,897 (6.8%)	16,678 (52.7%)	0.159 (0.040)	38.2 (5.6)
UK: Wales	71,828	02/06/10/14/18/22	22,592 (32.7%)	31,507 (46.7%)	5,283 (8.0%)	30,512 (45.4%)	0.122 (0.038)	42.6 (4.9)
USA	15,191	02/06/10	3,252 (22.0%)	6,500 (43.9%)	866 (5.8%)	6,838 (46.1%)	0.257 (0.008)	44.5 (4.7)
Total	1,268,220	02/06/10/14/18/22	342,776 (28.2%)	463,742 (37.6%)	81,307 (6.7%)	705,200 (57.4%)	0.140 (0.085)	35.9 (16.9)

### Measures at the country level

#### National‐level gender inequality

National‐level gender inequality was measured by the Gender Inequality Index (GII). This composite measure of gender inequality, developed by the United Nations Development Program (UNDP, 2021), is based on countries' scores on three dimensions: reproductive health (maternal mortality ratio and adolescent fertility rate), empowerment (proportion of parliamentary seats occupied by women versus men and educational attainment by gender), and the labour market (women's participation in the workforce) (UNDP, 2021). The GII ranges from 0 to 1, with higher values indicating greater levels of inequality.

#### Country wealth

Data on country wealth (i.e. gross domestic product [GDP] per capita; Atlas method, international dollars) were supplied by the World Bank Databank (2023) (World Bank, 2021). In our analyses, country wealth was included as a control variable at the country level given its correlation with national‐level gender inequality (*r* = −.73, *p* < .001; Table 2).

**Table 2:** Correlations between the variables included in the study (*n* = 234)

	1.	2.	3.	4.	5.	6.	7.	8.	9.	10.	11.	12.	13.
1. Survey cycle (year)	–												
2. Gender Inequality Index (GII)	−0.35*	–											
3. Country wealth (log GDP per cap), PPP (current intl $)	0.39*	−0.73*	–										
4. Some or a lot of schoolwork pressure (% boys)	0.07	0.07	−0.02	–									
5. Some or a lot of schoolwork pressure (% girls)	0.33*	−0.12	0.15*	0.89*	–								
6. Body dissatisfaction (% boys)	0.30*	−0.08	0.12	0.06	0.15*	–							
7. Body dissatisfaction (% girls)	0.29*	−0.36*	0.42*	−0.08	0.04	0.70*	–						
8. High classmate support (% boys)	0.03	−0.35*	0.13	−0.08	−0.10	−0.27*	−0.15*	–					
9. High classmate support (% girls)	−0.19*	−0.22*	0.09	−0.15*	−0.24*	−0.25*	−0.17*	0.91*	–				
10. 3–4 psychological symptoms (% boys)	0.31*	0.16*	0.04	0.27*	0.39*	0.39*	0.18*	−0.30*	−0.38*	–			
11. 3–4 psychological symptoms (% girls)	0.55*	−0.05	0.17*	0.29*	0.51*	0.36*	0.25*	−0.27*	−0.47*	0.88*	–		
12. Absolute gender gap in psychological	0.68*	−0.30*	0.29*	0.21*	0.49*	0.20*	0.25*	−0.15*	−0.42*	0.41*	0.79*	–	
13. Relative gender gap in psychological symptoms	0.47*	−0.41*	0.24*	0.06	0.24*	−0.11	0.08	0.11	−0.11	−0.29*	0.16*	0.70*	–

* *p* < 0.05.

### Measures at the individual level

#### Psychological symptoms

Adolescents reported the frequency with which they had experienced four psychological symptoms (i.e. feeling low; irritability or bad temper; feeling nervous; difficulties in getting to sleep) over the past 6 months, using the following response options: (a) ‘about every day’, (b) ‘more than once a week’, (c) ‘about every week’, (d) ‘about every month’, and (e) ‘rarely or never’. This instrument has demonstrated robust psychometric properties (Gariepy, McKinnon, Sentenac, & Elgar, 2016) and cross‐national invariance (Heinz et al., 2022). Each item was dichotomised to indicate whether the symptom was experienced frequently (at least weekly) versus infrequently (less than once a week), as per the international HBSC guidelines (Inchley et al., 2023). In order to capture a high symptom burden, combining the four items, a variable was created indicating 3 or 4 weekly psychological symptoms (1) versus 0 to 2 weekly psychological symptoms (0).

#### Gender

Adolescents were asked to indicate whether they are a boy or a girl.

#### Schoolwork pressure

Adolescents responded to the question, ‘How pressured do you feel by the schoolwork you have to do?’. The response options available were ‘not at all’ (1), ‘a little’ (2), ‘some’ (3), and ‘a lot’ (4). As per the international HBSC guidelines (Inchley et al., 2023), the responses were recoded to indicate high schoolwork pressure (some or a lot) (1) versus not at all or a little (0).

#### Body dissatisfaction

Adolescents were asked ‘Do you think your body is: Much too thin, A bit too thin, About the right size, A bit too fat or Much too fat’. This measure has been long used in the HBSC survey and has shown good test–retest stability (Geraets et al., 2023). A dummy variable was created in which the first and last response options were recoded as ‘dissatisfied with body (i.e, negative body image)’ (1) versus ‘satisfied with body’ (0).

#### Classmate support

Adolescents were asked to respond to three statements which examine the extent to which they experience support from their classmates (e.g. ‘The students in my class enjoy being together’). The response options available ranged from ‘strongly agree’ (1) to ‘strongly disagree’ (5). An average score over the 3‐item classmate support scale was calculated and transformed into a binary variable with 1 = high classmate support (average score of 4 or lower) and 0 = low classmate support (average score higher than 4). The cross‐country reliability and validity of this scale have been demonstrated in previous research (Torsheim et al., 2012).

#### Control variables

For each country/year group represented in the study, we calculated the weighted prevalence of 3–4 psychological symptoms, high schoolwork pressure, body dissatisfaction and classroom support while controlling for differences in age and family affluence [as measured with the relative Family Affluence Scale, FAS II; (Currie et al., 2008)]. Estimates of the absolute and relative gender gap in psychological symptoms were also controlled for age and family affluence (described below).

### Analytic plan

This study was pre‐registered (https://osf.io/7fqcv). STATA 16 was used to analyse the data. We used a meta‐regression approach whereby we first used weighted regressions at the individual level to estimate the absolute gender gap in psychological symptoms (i.e. the absolute difference between gender groups in the prevalence of experiencing 3 or 4 psychological symptoms at least weekly [%girls minus %boys]) and the relative gender gap (absolute gender difference divided by the mean, expressed as a percentage), whilst controlling for age and family affluence, and accounting for the clustering effect of schools. We ran these models separately in all 234 country/year groups represented in the sample.

In the second phase of the analysis, we used Prais‐Winsten linear regressions of absolute and relative gender differences in psychological symptoms. These models are similar to linear regressions but with panel‐corrected standard errors to account for serial dependence in the data. Our regressions included inverse variance weights based on the standard error (SE) of the absolute gender gap in psychological symptoms to minimise variance of the pooled average.

To answer RQ1, in which we assessed whether national‐level gender equality was associated with increases in the gender gap in psychological symptoms between 2002 and 2022 (also see Figure 1), we tested associations of survey cycle, the GII and (of main interest) the interaction between GII × survey cycle with the absolute and relative gender gap in psychological symptoms. If the interaction of the GII × survey cycle was significant, we plotted this interaction for interpretation. Next, to examine whether the interaction was primarily due to changing trends over time in psychological symptoms of boys or girls, we repeated the same analyses for boys and girls separately. If the interaction GII × survey cycle was found to be significant for a specific gender, this meant that, for that gender, trends over time in psychological symptoms were associated with national‐level gender‐equality. Significant interactions were plotted for interpretation.

To answer RQ2, we tested to what extent survey cycle, the GII and (of main interest) the interaction between GII × survey cycle predicted the hypothesised mediators (i.e. schoolwork pressure, body dissatisfaction and classmate support). We ran these analyses separately for boys and girls. We could not use the gender gap in the mediators as an outcome (as we did for psychological symptoms) as the direction of the gender gap for these mediators varied across countries. If the interaction GII × survey cycle was significant for a specific gender, this meant that, for that gender, trends over time in the mediator were associated with national‐level gender‐equality. Significant interactions were plotted for interpretation.

For those hypothesised mediators for which the GII × survey cycle interaction (and thus, path b in the conceptual model) was significant, we extended the models predicting the absolute and relative gender gap in psychological symptoms (from RQ1) to assess whether the GII × survey cycle interactions were altered after taking into account the mediators (and in particular, their interaction with survey cycle; RQ3). We then repeated the same analyses separately for boys and girls (just like for RQ1) to examine whether the observed interactions were primarily due to changing trends over time in the mediators in boys or in girls. If the interaction was found to be significant for a specific gender, then this meant that, for that gender, trends over time in the mediator explained the trends over time in psychological symptoms in gender‐equal countries.

## Results

### Descriptive statistics

**Figure 2:**
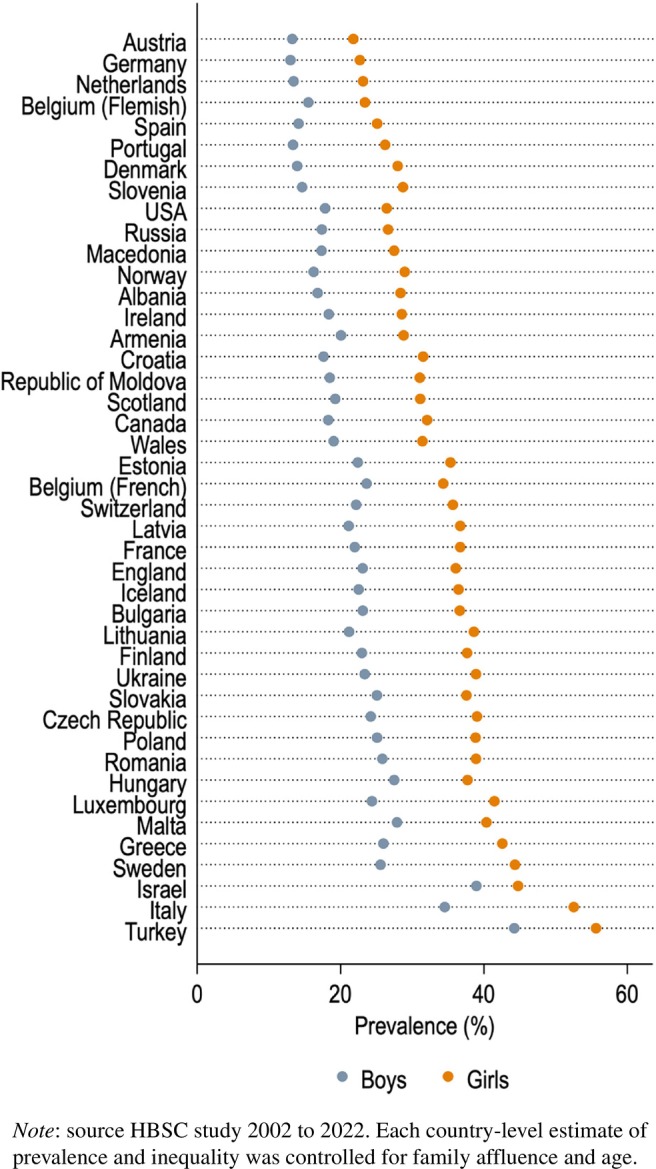
Prevalence of experiencing 3–4 psychological symptoms per week in 43 countries/regions.

Table 1 shows the variation among included countries in the prevalence of psychological symptoms, schoolwork pressure, body dissatisfaction and classmate support (across survey waves). In all countries, girls more frequently reported psychological symptoms than boys. However, prevalence rates and the size of the gender gap in psychological symptoms seemed to vary across countries (also see Figure 2).

**Figure 3:**
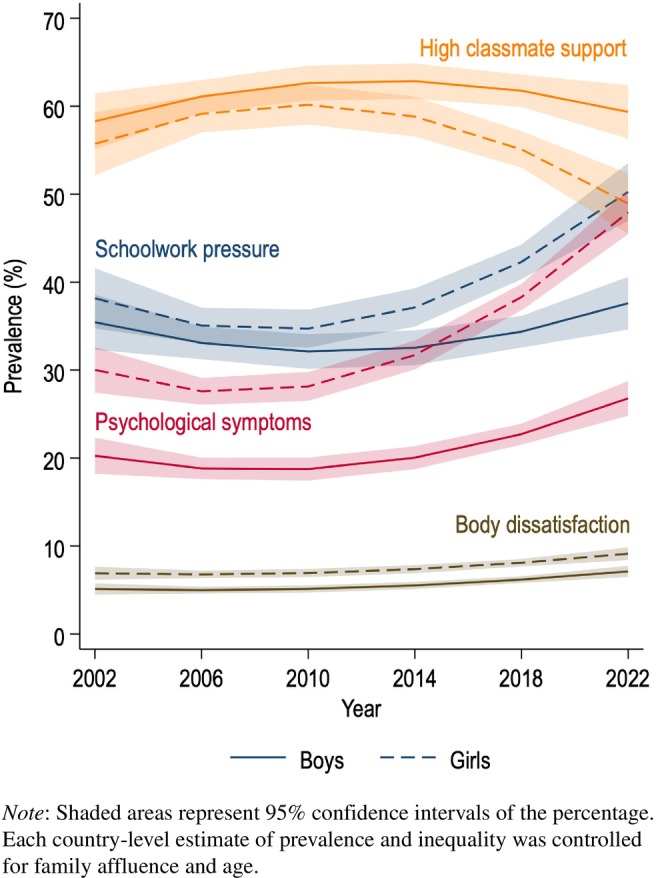
Prevalence of frequent psychological symptoms, schoolwork pressure, body dissatisfaction and high classmate support, by gender, in six survey cycles of the HBSC study.

Figure 3 shows gendered trends over time in the key variables of this study across countries. Across genders, over the 20‐year period, there was an increase in the percentage of adolescents that reported three or four psychological symptoms weekly, from 23.1% in 2002 to 37.7% in 2022 (not in figure). Increases over time were also observed in the percentage of adolescents reporting high levels of schoolwork pressure (from 34.8% in 2002 to 45.8% in 2022) and body dissatisfaction (from 5.9% in 2002 to 8.6% in 2022). The percentage of adolescents that reported high classmate support decreased from 56.7% in 2002 to 50.9% in 2022.

Pairwise correlations with survey year (Table 2) indicated that the changes over time in psychological symptoms, schoolwork pressure, body dissatisfaction and classmate support were different for boys and girls. While psychological symptoms increased over time for boys (*r* = .31, *p* < .001) and girls (*r* = .55, *p* < .001), the increase appears stronger for girls (as is also illustrated in Figure 3). Consequently, both absolute (*r* = .68, *p* < .001) and relative (*r* = .47, *p* < .001) gender gaps in psychological symptoms increased over time.

Table 2 also suggests that schoolwork pressure increased over time for girls (*r* = .33, *p* < .001) but not for boys; body dissatisfaction increased for both boys (*r* = .30, *p* < .001) and girls (*r* = .29, *p* < .001); and classmate support decreased for girls (*r* = −.19, *p* < .001) but not for boys. In line with these findings, Figure 3 suggests that the gender gap in schoolwork pressure as well as classmate support increased over time, with girls being worse off in both cases. Furthermore, Table 2 shows that psychological symptoms are correlated with high levels of schoolwork pressure, body dissatisfaction and low classmate support for boys as well as girls.

Among the countries in our sample, we observed a decline in the Gender Inequality Index (GII), from a mean of 0.18 (*SD* = 0.08) in 2002 to 0.10 (*SD* = 0.06) in 2022 (see Figure S1). Thus, over time, countries have become more gender‐equal. This is also shown by the pairwise correlation between the GII and survey cycle (Table 2; *r* = −.35, *p* < .001). Pairwise correlations also revealed a robust correlation between the GII and country wealth (*r* = −.73, *p* < .001). Finally, the GII was negatively related to both the absolute (*r* = −.30, *p* < .001) and relative (*r* = −.41, *p* < .001) gender gap in psychological symptoms.

### RQ1: National‐level gender equality and the increasing gender gap in psychological symptoms

Table 3 shows the results of the linear regression predicting the absolute and relative gender gap in adolescent psychological symptoms. With differences in country wealth and national‐level gender inequality controlled, the gender gap in psychological symptoms has increased over time (Model 1: *B* = 2.57, *p* < .001 for the absolute gender gap; *B* = 3.56, *p* < .001 for the relative gender gap). Figure 3 illustrates the increasing gender gap.

**Table 3:** Linear regression of absolute and relative gender differences in psychological symptoms (*n* = 234)

	Model 1			Model 2			Model 3			Model 4		
	*b*	95% CI	*p*	*b*	95% CI	*p*	*b*	95% CI	*p*	*b*	95% CI	*p*
1. Absolute gender gap in psychological symptoms												
Survey cycle (year)	2.57***	1.33, 3.81	.001	4.18***	2.63, 5.73	.001	1.26*	0.25, 2.28	.015	4.15***	1.88, 6.41	.001
Country wealth	−0.93	−3.11, 1.26	.405	−1.05	−2.94, 0.85	.279	−0.97	−2.74, 0.79	.279	−0.37	−1.58, 0.85	.556
GII	−5.86	−17.49, 5.76	.323	31.78**	8.16, 55.40	.008	12.54	−7.15, 32.24	.212	21.08*	2.37, 39.79	.027
GII × survey cycle				−12.13***	−17.52, −6.75	.001	−4.08*	−7.88, −0.28	.036	−8.85***	−12.78, −4.92	.001
Schoolwork pressure (% boys)							0.10	−0.25, 0.44	.585			
Schoolwork pressure (% girls)							−0.08	−0.47, 0.31	.694			
Schoolwork pressure (boys) × cycle							−0.11**	−0.20, −0.03	.005			
Schoolwork pressure (girls) × cycle							0.12**	0.04, 0.21	.005			
Classmate support (% boys)										0.24*	0.01, 0.48	.039
Classmate support (% girls)										−0.20	−0.45, 0.06	.127
Classmate support (boys) × cycle										0.05	−0.03, 0.12	.225
Classmate support (girls) × cycle										−0.08	−0.15, 0.00	.052
Intercept	13.89	−9.38, 37.16	.242	9.57	−12.46, 31.60	.395	13.83	−5.65, 33.32	.164	3.58	−12.80, 19.97	.668
*R* ^2^	.74			.78			.86			.86		
*ρ*	0.16			0.18			0.28			0.19		
2. Relative gender gap in psychological symptoms												
Survey cycle (year)	3.56***	1.78, 5.33	.001	5.70***	3.73, 7.66	.001	4.86**	1.93, 7.78	.001	7.36**	1.93, 12.78	.008
Country wealth	−6.74**	−11.73, −1.75	.008	−6.81**	−11.78, −1.85	.007	−6.62**	−11.08, −2.17	.004	−4.48*	−8.18, −0.79	.017
GII	−68.35***	−102.63, −34.07	.001	−17.87	−62.45, 26.71	.432	−34.45	−74.31, 5.41	.090	−13.93	−52.93, 25.08	.484
GII × survey cycle				−16.21***	−24.26, −8.16	.001	−9.50*	−18.21, −0.79	.033	−14.63***	−22.49, −6.77	<.001
Schoolwork pressure (% boys)							−0.29	−1.55, 0.96	.647			
Schoolwork pressure (% girls)							0.43	−0.74, 1.60	.471			
Schoolwork pressure (boys) × cycle							−0.04	−0.32, 0.24	.768			
Schoolwork pressure (girls) × cycle							0.01	−0.25, 0.27	.934			
Classmate support (% boys)										1.32***	0.66, 1.97	.001
Classmate support (% girls)										−1.13***	−1.74, −0.51	.001
Classmate support (boys) × cycle										−0.17	−0.41, 0.07	.161
Classmate support (girls) × cycle										0.13	−0.09, 0.34	.237
Intercept	113.37***	60.19, 166.56	.001	106.67***	52.67, 160.67	.001	103.63***	54.25, 153.02	.001	70.07***	31.98, 108.17	.001
*R* ^2^	.83			.84			.84			.83		
*ρ*	0.26			0.27			0.26			0.20		

*Note*: Shown are the slope coefficient, 95% confidence interval (CI) and *p*‐value from country/year‐level Prais‐Winsten linear regressions. ρ(rho) represents the autocorrelation parameter; Gender Inequality Index (GII); controlled for age and family affluence and accounting for clustering effect of schools.

*p < .05. **p < .01. ***p < .001.

**Figure 4:**
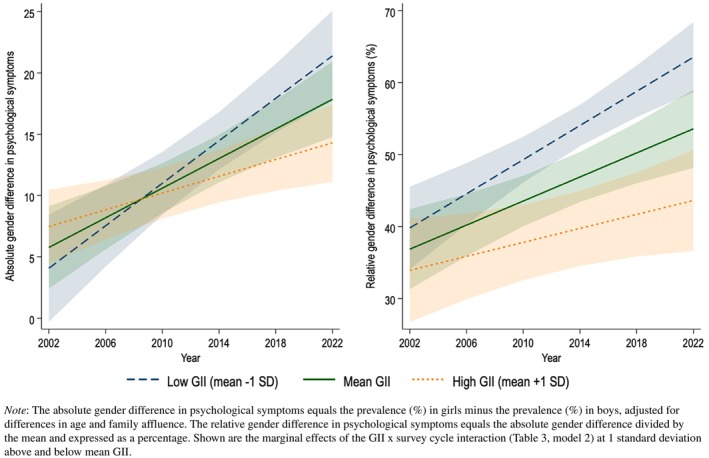
Simple effects of the Gender Inequality Index (GII) on absolute (right) and relative (left) gender differences in the prevalence of experiencing 3–4 psychological symptoms per week across 43 countries/regions, 2002‐2022.

The interaction GII × survey cycle was significant, showing that the gender gap in psychological symptoms has increased less over time in more gender‐unequal countries (Model 2: *B* = −12.13, *p* < .001 for the absolute gender gap; *B* = −16.21, *p* < .001 for the relative gender gap). Or, phrased alternatively, the gender gap in psychological symptoms has increased more in more gender‐equal countries. Figure 4 illustrates this: across countries, absolute and relative gender differences in psychological symptoms have increased over time, but the increase is stronger in more gender‐equal (low GII) countries.

**Figure 5:**
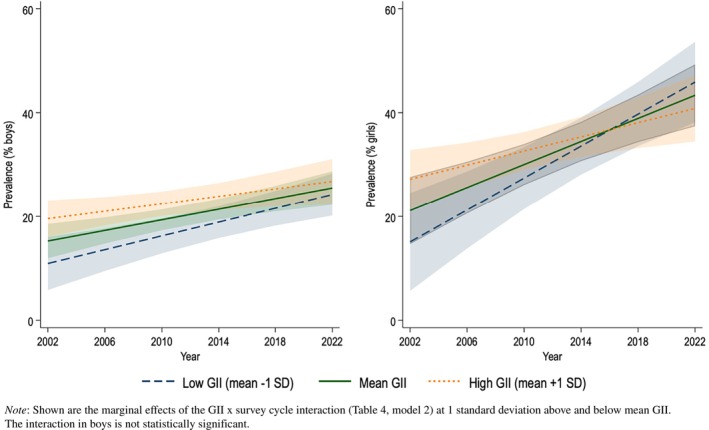
Simple effects of the Gender Inequality Index on the prevalence of 3–4 weekly psychological symptoms in boys (left) and girls (right) across 43 countries/regions, 2002‐2022.

When repeating the analyses for boys and girls separately (see Table S1), results indicate that the association between the GII and psychological symptoms changed over time for girls (*b* = −19.86, *p* = .004) but not for boys (*b* = −7.07, *p* = .117). This result is illustrated in Figure 5: For girls, the prevalence of psychological symptoms overall increased, but this increase was larger in more gender‐equal (low GII) countries. While in 2002, psychological symptoms seemed *lower* among girls in more gender‐equal countries, in 2022 psychological symptoms among girls seemed *higher* in more gender‐equal countries. For boys this was not the case; across survey waves, national‐level gender equality was associated with fewer psychological symptoms.

### RQ2: National‐level gender equality and changes over time in schoolwork pressure, body dissatisfaction and classmate support among boys and girls

**Figure 6:**
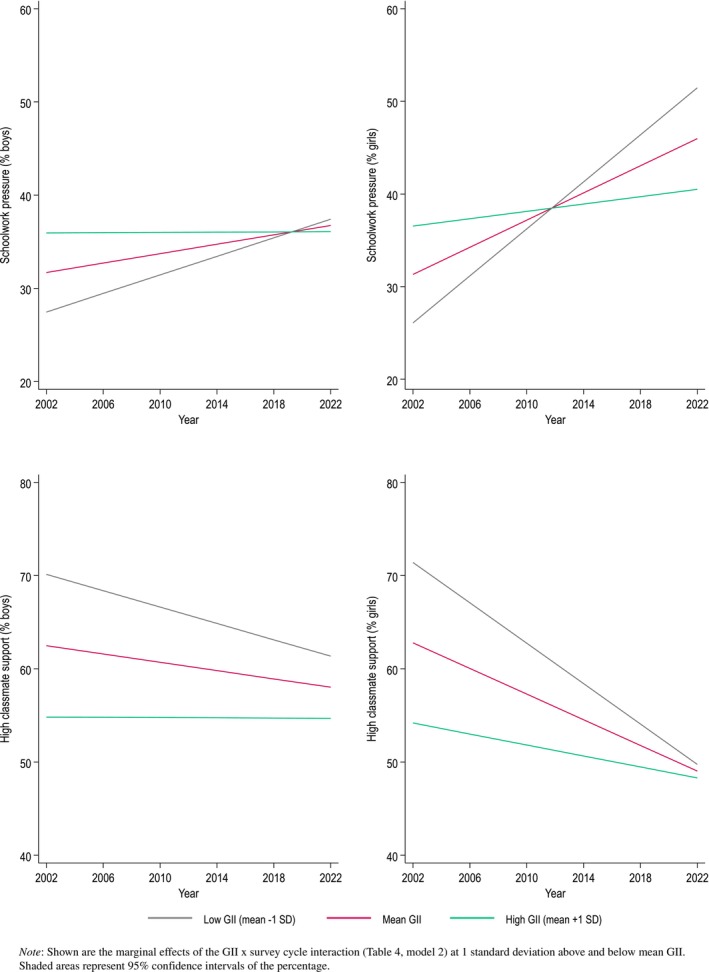
Simple effects of the Gender Inequality Index (GII) on the prevalence of schoolwork pressure and high classmate support in boys (left) and girls (right) across 43 countries/regions, 2002‐2022.

Table 4 shows that for girls, schoolwork pressure increased less over time in gender‐unequal countries as compared to more gender‐equal countries (GII × survey cycle: *b* = −24.78, *p* < .001). Figure 6 illustrates this effect: while in 2002, schoolwork pressure was higher among girls in more gender‐unequal (high GII) countries, in 2022 schoolwork pressure was higher among girls in more gender‐equal countries. For boys, this interaction effect was also found, but it was less strong (GII × survey cycle: *b* = −11.36, *p* < .001).

**Table 4:** Linear regression of three mediators: schoolwork pressure, body dissatisfaction and classmate support (*n* = 234)

	Schoolwork pressure			Body dissatisfaction			Classmate support		
	*b*	95% CI	*p*	*b*	95% CI	*p*	*b*	95% CI	*p*
1. Boys									
Country wealth	2.04	−0.84, 4.91	.164	0.31	−0.47, 1.09	.435	−3.78	−8.14, 0.58	.090
Survey cycle (year)	2.66***	1.63, 3.69	.001	0.30	−0.08, 0.68	.121	−2.34**	−4.04, −0.65	.007
GII (time varying)	60.46***	30.01, 90.90	.001	−1.70	−9.03, 5.63	.649	−98.57***	−143.09, −54.06	.001
GII × cycle	−11.36***	−18.10, −4.61	.001	0.65	−1.38, 2.67	.532	10.00*	0.38, 19.63	.042
Intercept	0.80	−32.07, 33.67	.962	1.39	−7.02, 9.80	.746	116.82***	69.63, 164.00	.001
*R* ^2^	.86			.78			.95		
*ρ*	0.60			0.51			0.51		
2. Girls									
Country wealth	0.53	−3.30, 4.37	.786	0.81	−0.28, 1.89	.144	−1.17	−6.34, 4.01	.659
Survey cycle (year)	6.53***	4.70, 8.36	.001	0.24	−0.44, 0.92	.485	−5.41***	−7.82, −3.00	.001
GII (time varying)	85.25***	43.77, 126.74	.001	−6.34	−17.36, 4.69	.260	−117.94***	−170.91, −64.97	.001
GII × cycle	−24.78***	−34.04, −15.51	.001	0.07	−2.81, 2.96	.961	18.26**	6.28, 30.25	.003
Intercept	10.49	−34.34, 55.32	.646	−0.67	−12.21, 10.86	.909	94.76***	38.51, 151.00	.001
*R* ^2^	.87			.82			.92		
*ρ*	0.57			0.42			0.49		

Shown are the slope coefficient, 95% confidence interval (CI) and *p*‐value from country/year‐level Prais‐Winsten linear regressions. ρ(rho) represents the autocorrelation parameter. Gender Inequality Index (GII); controlled for age and family affluence and accounting for clustering effect of schools.

*p < .05; **p < .01; ***p < .001.

Table 4 also shows that for girls, classmate support declined less in gender‐unequal countries as compared to more gender‐equal countries (GII × survey cycle: *b* = 18.26, *p* = .003). While classmate support among girls in 2002 was higher in gender‐equal (low GII) countries, in 2022 levels of classmate support did not differ anymore by GII (see Figure 6). For boys, across survey cycles, levels of classmate support were higher in more gender‐equal countries, however, the difference in classmate support between gender‐equal and gender‐unequal countries became smaller (GII × cycle: *b* = 10.00, *p* = .042).

For body dissatisfaction, the interaction between GII × survey cycle was not significant for both genders. This implies that, for boys as well as girls, trends over time in body dissatisfaction did not differ between gender‐equal and gender‐unequal countries.

### RQ 3: Explaining the increasing gender gap in psychological symptoms in more gender‐equal countries

In RQ3, we examined to what extent the stronger increase in the gender gap in adolescent psychological symptoms in gender‐equal countries can be explained by changes over time in schoolwork pressure and classmate support in boys and girls in these countries. Model 3 in Table 3 suggests that trends in schoolwork pressure in girls partly explain the larger increase in the absolute gender gap in adolescent psychological symptoms in more gender‐equal countries. This conclusion is based on the significant ‘schoolwork pressure (girls) × survey cycle’ interaction and the decline in the coefficient of the ‘GII × survey cycle’ interaction in Model 3, as compared to Model 2 (from *b* = −12.13, *p* < .001 in Model 2 to *b* = −4.08, *p* = .036 in Model 3). For boys, the interaction was also significant but had an opposite direction, meaning that a stronger increase in schoolwork pressure over time was associated with a smaller gender gap in psychological symptoms. When repeating the analyses for boys and girls separately (see Table S1), results indicate that for girls, the stronger increase in schoolwork pressure in more gender‐equal countries partly explained the stronger increase in psychological symptoms in these countries (*b* of ‘schoolwork pressure × survey cycle’ interaction on psychological symptoms = 0.09, *p* = .007; the coefficient of the GII × survey cycle interaction declined from *b* = −19.86, *p* = .004 in Model 2 to *b* = −10.22, *p* = .037 in Model 3). Thus, the increased absolute gender gap in psychological symptoms in more gender‐equal countries was partly explained by a stronger increase in schoolwork pressure over time among girls in those countries.

Model 4 in Table 3 shows that the gendered trends over time in classmate support cannot explain the stronger increase in the gender gap in adolescent psychological symptoms in more gender‐equal countries. The interactions between classmate support and survey cycle were not significantly associated with either the absolute or relative gender gap in psychological symptoms. When repeating the analyses for boys and girls separately (see Table S1), results showed that for both boys and girls, the interaction of ‘classmate support × survey cycle’ on psychological symptoms was significant (*b* = −0.04, *p* = .010 for boys and *b* = −0.09, *p* = .005 for girls). However, the coefficient for the ‘GII × survey cycle’ interaction hardly changes after adding the interactions. Therefore, the increasing gender gap in psychological symptoms in more gender‐equal countries could not be explained by gendered trends in classmate support.

## Discussion

Adolescent mental health is a public health concern worldwide. In recent years, internalising problems have increased among adolescent girls in particular, resulting in a widening gender gap across (Western) countries. However, countries differ in the extent to which this gender gap has grown (Cosma et al., 2023). The current study examined whether national‐level gender‐equality was associated with increases in the gender gap in psychological symptoms between 2002 and 2022 and if so, whether these increases could be explained by changes over time in the experience of social stressors (i.e. more schoolwork pressure and body dissatisfaction and lower classmate support) among boys and girls in more gender‐equal countries.

Our results showed that the absolute and relative gender gap in psychological symptoms between 2002 and 2022 indeed increased to a larger extent in more gender‐equal countries. This increasing gender gap in more gender‐equal countries was mainly due to a larger over‐time increase in psychological symptoms among girls in these countries. Moreover, we found less favourable time trends for schoolwork pressure and classmate support in more gender‐equal countries. Although this applied to both boys and girls, this was more strongly the case for girls. Finally, our results indicated that the larger increase in schoolwork pressure among girls in more gender‐equal countries may partly explain the increased absolute gender gap in psychological symptoms in more gender‐equal countries in the last two decades.

An alternative way of describing the results of this study is that the direction of the association between national‐level gender equality and schoolwork pressure, classmate support and (the gender gap in) psychological symptoms changed over time. In the early 2000s, girls in more gender‐equal countries were doing better in terms of psychological symptoms, schoolwork pressure and classmate support as compared to their peers in less gender‐equal countries, but they lost this advantage in the 2010s. For schoolwork pressure and classmate support a similar, yet less strong, trend was observed among the boys. These results are in line with our observation that studies using less recent data (de Looze et al., 2018; Torsheim et al., 2006; Viner et al., 2012) revealed better mental health among adolescents in more gender‐equal countries, while studies using more recent data showed the opposite for boys and girls (Campbell et al., 2021) or only for girls (Guo et al., 2022).

The change in the direction of associations with national‐level gender equality over time may be explained by changes in the wider societal context during this period, which may act as confounders in the association between gender equality and adolescent mental health. Between 2002 and 2022, most countries included in our study have not only become more gender‐equal, they have also become increasingly competitive, individualistic, perfectionist and digital (Boer et al., 2023; Chassiakos, Linda, & Stager, 2020; Curran & Hill, 2022; Santos, Varnum, & Grossmann, 2017). This may have resulted in a context in which adolescents feel they are more ‘on their own’: they perceive more pressure to succeed, are more self‐critical and feel more responsible for their own failures and mistakes. In this context, the positive effects of national‐level gender equality (e.g. more opportunities) may have become overshadowed by increasing pressure to ‘do it all’ and ‘be perfect all the time’ for girls (Strömbäck, Formark, Wiklund, & Malmgren‐Olsson, 2014). Girls may suffer from the ‘superwoman’ ideal in which they are meant to succeed academically, occupationally and financially as well as retain the more traditional gendered expectations (Landstedt, Asplund, & Gillander Gådin, 2009; Wiklund, Bengs, Malmgren‐Olsson, & Öhman, 2010). The occurrence of the COVID‐19 pandemic in 2020–2021, with related school closures, social distancing measures and worries about one's own health and that of others, may have intensified the experience of these stressors among girls in particular.

Another potential explanation for our finding that gender equality is associated with higher, rather than lower, levels of psychological symptoms for girls in more recent years is that girls in more gender‐equal countries in 2022 may be more conscious (as compared to 2002) of the existing inequalities and ongoing gender‐discrimination that women still face (UNDP, 2021), which may negatively impact their mental health (Demkowicz et al., 2023). In the early 2000s, there may have been a false excitement, optimism or feeling of victory, as women appreciated equality and the possibilities of new opportunities. As time passed, the reality of the double burden may have become more tangible, as gender equality may have been understood to be one‐sided. In other words, the mismatch between the ideal of gender equality and the lived experiences of girls and women in more (but still not even close to fully) gender‐equal societies may be disillusioning for women and girls living in these societies. This may be especially true for younger generations, considering their evolving perspectives on equality and fairness (Arsenio & Willems, 2017) and involvement in social activism (Wallis & Loy, 2021). In sum, the negative association of gender equality with adolescents' and especially girls'‐ mental health, in recent years is unlikely to reflect a direct negative effect of national‐level gender equality on adolescent mental health. Rather, it may reflect the occurrence of other societal trends that are more likely to take place in more gender‐equal countries and young people's frustration with partial and incomplete gender equality.

### Strengths and limitations

This study has a number of strengths, such as the use of large and nationally representative data sets across 43 countries, a trend analysis over a relatively long time period of 20 years, and a standard protocol for the data collection across samples. Some limitations should however be considered when interpreting our results, pointing to avenues for further research. First, whilst our study design precluded tests of causal mediation by schoolwork pressure, body dissatisfaction and classmate support, given the aggregated and repeated cross‐sectional nature of the data, causality pathways cannot be inferred. Particularly, we suggest that trends in schoolwork pressure among girls may contribute to explaining the trends in psychological symptoms in more gender equal countries. However, given the nature of our data it may also be the other way around.

Second, while our results may reflect an effect of national‐level gender equality, there may be other national‐level variables which are associated with national‐level gender equality, that explain the increase in (the gender gap in) psychological symptoms in more gender‐equal countries. Research for example showed that gender‐equal countries are increasingly individualistic (Santos et al., 2017), hold more meritocratic beliefs (Weinberg et al., 2021) or experience increasing levels of perfectionism (Curran & Hill, 2019). Due to limited data availability across countries, these variables were not included in our study. Also, as ethnicity was not included as a mandatory variable in the HBSC survey, we could not control for potential shifts in the composition of the adolescent population. This may have impacted our findings as minority status, including ethnic group minority, relates to young people's mental health, especially during the years of the pandemic (Eboigbe, Simon, Wang, & Tyrell, 2023). Third, while psychological symptoms represent an important aspect of mental health, other aspects of mental health, such as life satisfaction, depressive symptoms and anxiety were not examined. Furthermore, our measure of body dissatisfaction was limited to perceptions of feeling too thin or too fat, which may be more relevant to girls' evaluation of their bodies and did not take into account other aspects of body dissatisfaction, such as being muscular, which have been shown to play a greater role in boys' body image (Baker et al., 2019). Finally, while gender differences are central to this study, our measure of gender (‘are you a boy or a girl?’) does not reflect the experience of young people whose gender identity does not match these binary categories, nor those for whom the sex assigned at birth does not correspond with their gender identity.

### Implications

The increasing psychological symptoms in the last two decades and more specifically, the increase in the gender gap in these symptoms in more gender equal societies, reinforce public concerns on this topic. Our findings suggest a need for increased awareness among adolescents, their parents and society at large of these problems and for the introduction of policies and interventions which may decrease the levels of stressors on young women in more gender‐equal societies. Our findings also urge for more research to replicate our findings in more countries and to better understand the mechanisms behind it.

## Conclusion

In recent years, societal concerns on adolescent mental health have increased due to increasing internalising problems, especially among girls. This study showed that the gender gap in psychological symptoms between 2002 and 2022 indeed increased to a larger extent in more gender‐equal countries, mainly due to a larger over‐time increase in psychological symptoms among girls in these countries. A larger increase in schoolwork pressure among girls in gender‐equal countries may explain this trend. Results lend support to the idea that gender equality may place a ‘double burden’ on adolescent girls. However, far from advocating that gender equality is a negative situation, these findings suggest that much work remains to achieve full gender equality, where men and women really share the burdens and stressors and have equal opportunities in everyday life.

**Key points**

- In the period 2002–2022, across 43 countries, psychological symptoms increased among adolescents, especially in girls, resulting in a wider gender gap.
- The gender gap in psychological symptoms increased more in gender‐equal countries, as compared to gender‐unequal countries.
- Between 2002 and 2022, boys and especially girls in more gender‐equal countries reported larger increases in schoolwork pressure and larger declines in classmate support, as compared to boys and girls in less gender‐equal countries.
- The rise in schoolwork pressure among girls in more gender‐equal countries may partly explain the widening gender gap in psychological symptoms in these countries.
- The increase in the experience of psychological symptoms and schoolwork pressure and the decline in classmate support among adolescent boys and particularly girls in more gender‐equal countries demands a practice and research response in both genders.

## Supporting information


**Table S1**. Linear regression of the prevalence of 3 to 4 weekly psychological symptoms (*n* = 234)
**Figure S1**. Gender Inequality Index (GII) in 43 HBSC countries and regions, 2002 to 2022.

## Data Availability

The data that support the findings of this study are openly available in repository of the University of Bergen, Norway, at https://www.uib.no/en/hbscdata/113290/open‐access. The 2022 data will become available in 2026.
