# Supplementary material for: Exploring mechanisms behind the increasing gender gap in adolescent psychological symptoms, 2002–2022: the role of national‐level gender equality
Source: J Child Psychol Psychiatry. 2024 Dec 7;66(5):737–51. doi: 10.1111/jcpp.14081 (PMC12018292; doi:10.1111/jcpp.14081)
Supplement: Supplementary file 1 — Table S1. Linear regression of the prevalence of 3 to 4 weekly psychological symptoms (n = 234) Figure S1. Gender Inequality Index (GII) in 43 HBSC countries and regions, 2002 to 2022. [file JCPP-66-737-s001.docx]

**Supporting Information**

**Table S1**

*Linear regression of the prevalence of 3 to 4 weekly psychological symptoms (n=234)*

|  | | Model 1 | | | | | | Model 2 | | | | | | Model 3 | | | | | | Model 4 | | | | | |
| --- | --- | --- | --- | --- | --- | --- | --- | --- | --- | --- | --- | --- | --- | --- | --- | --- | --- | --- | --- | --- | --- | --- | --- | --- | --- |
|  | | b | | 95% CI | | p | | b | | 95% CI | | p | | b | | 95% CI | | p | | b | | 95% CI | | p | |
|  | | *1. Three to four psychological symptoms (% boys)* | | | | | | | | | | | | | | | | | | | | | | | |
| Survey cycle (year) | | 2.16*** | | 0.96,3.36 | | <0.001 | | 3.08*** | | 1.36,4.80 | | <0.001 | | 1.21 | | -0.39,2.81 | | 0.139 | | 5.57*** | | 2.50,8.64 | | <0.001 | |
| Country wealth | | 1.95 | | -0.51,4.40 | | 0.120 | | 2.06 | | -0.12,4.25 | | 0.064 | | 1.77 | | -0.51,4.05 | | 0.129 | | 1.17 | | -0.71,3.04 | | 0.224 | |
| GII | | 34.69*** | | 15.21,54.16 | | <0.001 | | 57.04** | | 16.86,97.21 | | 0.005 | | 48.24* | | 11.42,85.06 | | 0.010 | | 48.26* | | 5.75,90.77 | | 0.026 | |
| GII x survey cycle | |  | |  | |  | | -7.07 | | -15.92,1.78 | | 0.117 | | -5.15 | | -12.87,2.58 | | 0.192 | | -7.64 | | -16.66,1.37 | | 0.097 | |
| Schoolwork pressure (% boys) | |  | |  | |  | |  | |  | |  | | 0.04 | | -0.14,0.22 | | 0.693 | |  | |  | |  | |
| Schoolwork pressure (boys) x cycle | |  | |  | |  | |  | |  | |  | | 0.04 | | -0.01,0.09 | | 0.086 | |  | |  | |  | |
| Classmate support (% boys) | |  | |  | |  | |  | |  | |  | |  | |  | |  | | 0.00 | | -0.14,0.13 | | 0.943 | |
| Classmate support (boys) x cycle | |  | |  | |  | |  | |  | |  | |  | |  | |  | | -0.04** | | -0.08,-0.01 | | 0.010 | |
| Intercept | | -11.96 | | -37.97,14.04 | | 0.367 | | -16.45 | | -42.46,9.56 | | 0.215 | | -12.72 | | -39.44,14.01 | | 0.351 | | -4.99 | | -32.75,22.77 | | 0.725 | |
| R^2^ | | 0.83 | |  | |  | | 0.84 | |  | |  | | 0.85 | |  | |  | | 0.85 | |  | |  | |
| *ρ* | | 0.47 | |  | |  | | 0.46 | |  | |  | | 0.48 | |  | |  | | 0.46 | |  | |  | |
|  | |  | |  | |  | |  | |  | |  | |  | |  | |  | |  | |  | |  | |
|  | | *2. Three to four psychological symptoms (% girls)* | | | | | | | | | | | | | | | | | | | | | | | |
| Survey cycle (year) | | 4.71*** | | 2.31,7.11 | | <0.001 | | 7.33*** | | 4.09,10.57 | | <0.001 | | 1.29 | | -1.50,4.09 | | 0.365 | | 11.37*** | | 6.17,16.57 | | <0.001 | |
| Country wealth | | 0.65 | | -3.87,5.16 | | 0.779 | | 0.65 | | -3.16,4.45 | | 0.739 | | 0.29 | | -3.57,4.15 | | 0.885 | | -0.57 | | -3.35,2.21 | | 0.688 | |
| GII | | 27.92* | | 3.52,52.32 | | 0.025 | | 89.83** | | 29.80,149.85 | | 0.003 | | 61.47* | | 8.04,114.91 | | 0.024 | | 68.05* | | 7.78,128.32 | | 0.027 | |
| GII x survey cycle | |  | |  | |  | | -19.86** | | -33.18,-6.55 | | 0.004 | | -10.22* | | -19.81,-0.63 | | 0.037 | | -18.57** | | -31.32,-5.83 | | 0.004 | |
| Schoolwork pressure (% girls) | |  | |  | |  | |  | |  | |  | | -0.01 | | -0.28,0.26 | | 0.947 | |  | |  | |  | |
| Schoolwork pressure (girls) x cycle | |  | |  | |  | |  | |  | |  | | 0.09** | | 0.03,0.16 | | 0.007 | |  | |  | |  | |
| Classmate support (% girls) | |  | |  | |  | |  | |  | |  | |  | |  | |  | | 0.04 | | -0.21,0.29 | | 0.757 | |
| Classmate support (girls) x cycle | |  | |  | |  | |  | |  | |  | |  | |  | |  | | -0.09** | | -0.16,-0.03 | | 0.005 | |
| Intercept | | 6.09 | | 42.03,54.20 | | 0.804 | | -3.05 | | -48.86,42.76 | | 0.896 | | 8.34 | | -35.09,51.77 | | 0.707 | | 13.54 | | -30.53,57.61 | | 0.547 | |
| R^2^ | | 0.85 | |  | |  | | 0.87 | |  | |  | | 0.90 | |  | |  | | 0.90 | |  | |  | |
| *ρ* | | 0.33 | |  | |  | | 0.35 | |  | |  | | 0.42 | |  | |  | | 0.35 | |  | |  | |

*p<0.05. **p<0.01. ***p<0.001.

*Note:* Shown are the slope coefficient, 95% confidence interval (CI) and p-value from country/year-level Prais-Winsten linear regressions. ρ(rho) represents the autocorrelation parameter. Gender Inequality Index (GII); controlled for age and family affluence and accounting for clustering effect of schools.

**Figure S2**

*Gender Inequality Index (GII) in 43 HBSC countries and regions, 2002 to 2022*


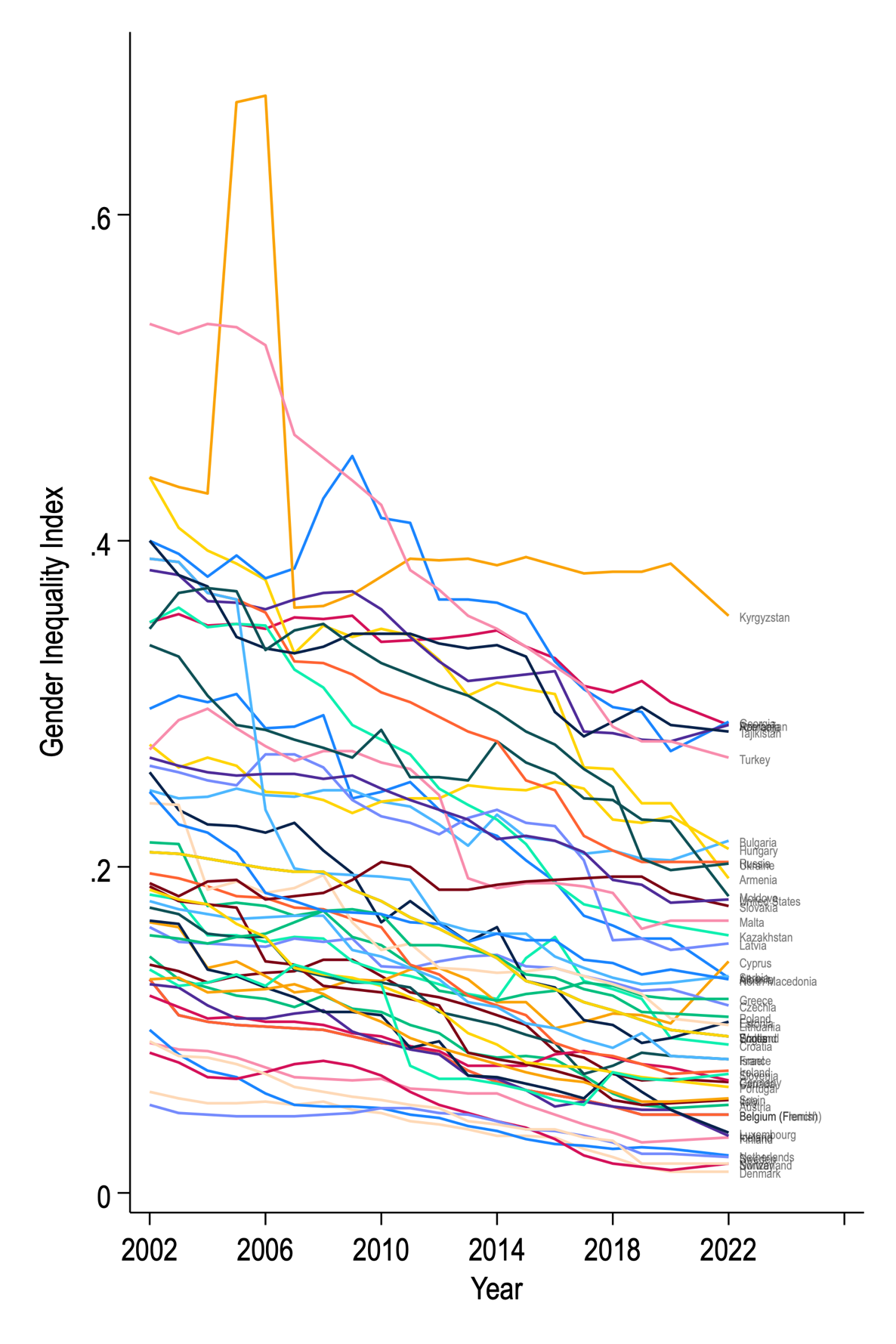


*Note:* Source: UNDP, 2022.
